# An Adult Patient With a Tetralogy of Fallot Case

**DOI:** 10.7759/cureus.11658

**Published:** 2020-11-23

**Authors:** Wail Alkashkari, Faisal Al-Husayni, Ahmed Almaqati, Jamilah AlRahimi, Saad Albugami

**Affiliations:** 1 Cardiology, King Abdullah International Medical Research Center, King Saud bin Abdulaziz University for Health Sciences, Jeddah, SAU; 2 Cardiology, King Faisal Cardiac Center, King Abdulaziz Medical City, Ministry of National Guard Health Affairs, Jeddah, SAU; 3 Internal Medicine, King Abdulaziz Medical City, Ministry of National Guard Health Affairs, Jeddah, SAU; 4 Internal Medicine, King Abdullah International Medical Research Center, King Saud bin Abdulaziz University for Health Sciences, Jeddah, SAU; 5 Internal Medicine, King Fahad Armed Forces Hospital, Jeddah, SAU; 6 Cardiology, Echocardiography, King Faisal Cardiac Center, King Abdulaziz Medical City, Ministry of National Guard Health Affairs, King Saud bin Abdulaziz University for Health Sciences, King Abdullah International Medical Research Center, Jeddah, SAU

**Keywords:** tetralogy of fallot, adult, cardiac surgical procedure, congenital heart defects, heart septal defects, murmur, echocardiogram, tof

## Abstract

Tetralogy of Fallot (ToF) is considered the most frequent cyanotic congenital heart abnormality with a low adulthood survival rate if kept untreated. The majority of cases are symptomatic during infancy and mandate early treatment. Few instances of survival to asymptomatic middle-age patients have been reported, and they are decreasing due to early detection. We reported a case of a middle-aged man who was asymptomatic during his life and recently diagnosed with ToF. The patient underwent surgical repair with excellent outcomes. The case represents the possibility of diagnosing such cases in a relatively old patient despite medical development and advances.

## Introduction

Tetralogy of Fallot (ToF), which composes right ventricular outflow tract (RVOT) obstruction, ventricular septal defect, overriding aorta, and concentric right ventricular hypertrophy, is considered the most frequent cyanotic congenital heart abnormality [1]. The severity of RVOT stenosis and pulmonary artery anatomy profoundly affect the degree of cyanosis. Etienne-Louis Fallot first described it in 1888 [2].

ToF may present with different phenotypes ranging from mild to severe cases such as Fallot-type double outlet right ventricle or ToF with pulmonary atresia [3]. Management approaches differ based on severity. Prognosis of ToF has improved significantly after surgical correction with the survival of up to 25 years post-repair, making surgical intervention the treatment of choice on ToF [4,5]. However, delaying the diagnosis and late intervention are highly associated with poor outcomes [6]. 

Diagnosing ToF is becoming easier with technological advances. Once a patient is suspected of having ToF, electrocardiogram (ECG) and chest x-ray (CXR) must be obtained. Right axis deviation, right ventricular hypertrophy signs, and prominent R waves in anterior precordial leads with large S waves in the lateral precordial leads suggest ToF [7]. CXR may present right ventricular apex displacement due to right ventricular hypertrophy, while the hypoplastic pulmonary outflow tract offers as mediastinal shadow curtailing [7]. This image forms a boot-shaped cardiac silhouette, a signature finding in ToF cases. Nonetheless, echocardiogram (ECHO) is the gold standard to confirm the diagnosis of ToF [7].

With the luxury of possessing diverse diagnostic tools, diagnosis of ToF mostly occurs during childhood. In contrast, we describe a case of late ToF diagnosis in an adult patient.

## Case presentation

**Figure 1:**
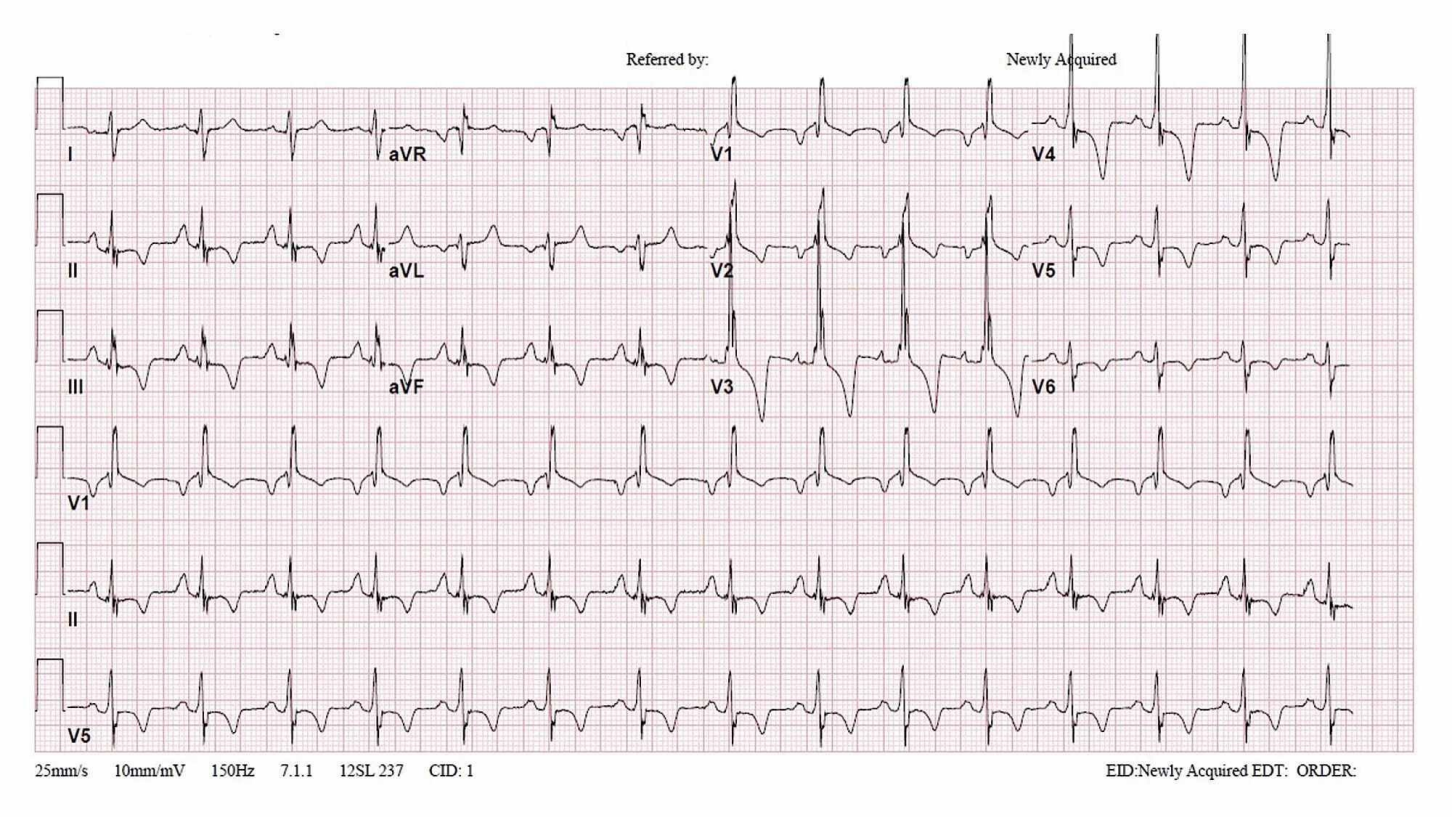
Patient’s electrocardiogram showing right bundle branch block and right ventricular hypertrophy strain pattern.

A 29-year-old gentleman with an illness-free past medical history presented with dyspnea. The patient was doing well throughout his life until a week before his presentation when he started to experience exertional dyspnea with moderate activities associated with atypical pricking chest pain. The patient was capable of lying flat, and his vitals were normal apart from an approximately blood pressure of 150/90 mmHg in all limbs. Physical examination revealed an elevated jugular venous pressure and a harsh systolic ejection murmur best heard at the left upper sternal border. Basic blood tests were normal; however, ECG exhibited a right bundle branch block (RBBB) and right ventricular hypertrophy (RVH) strain pattern (Figure 1).

**Figure 2:**
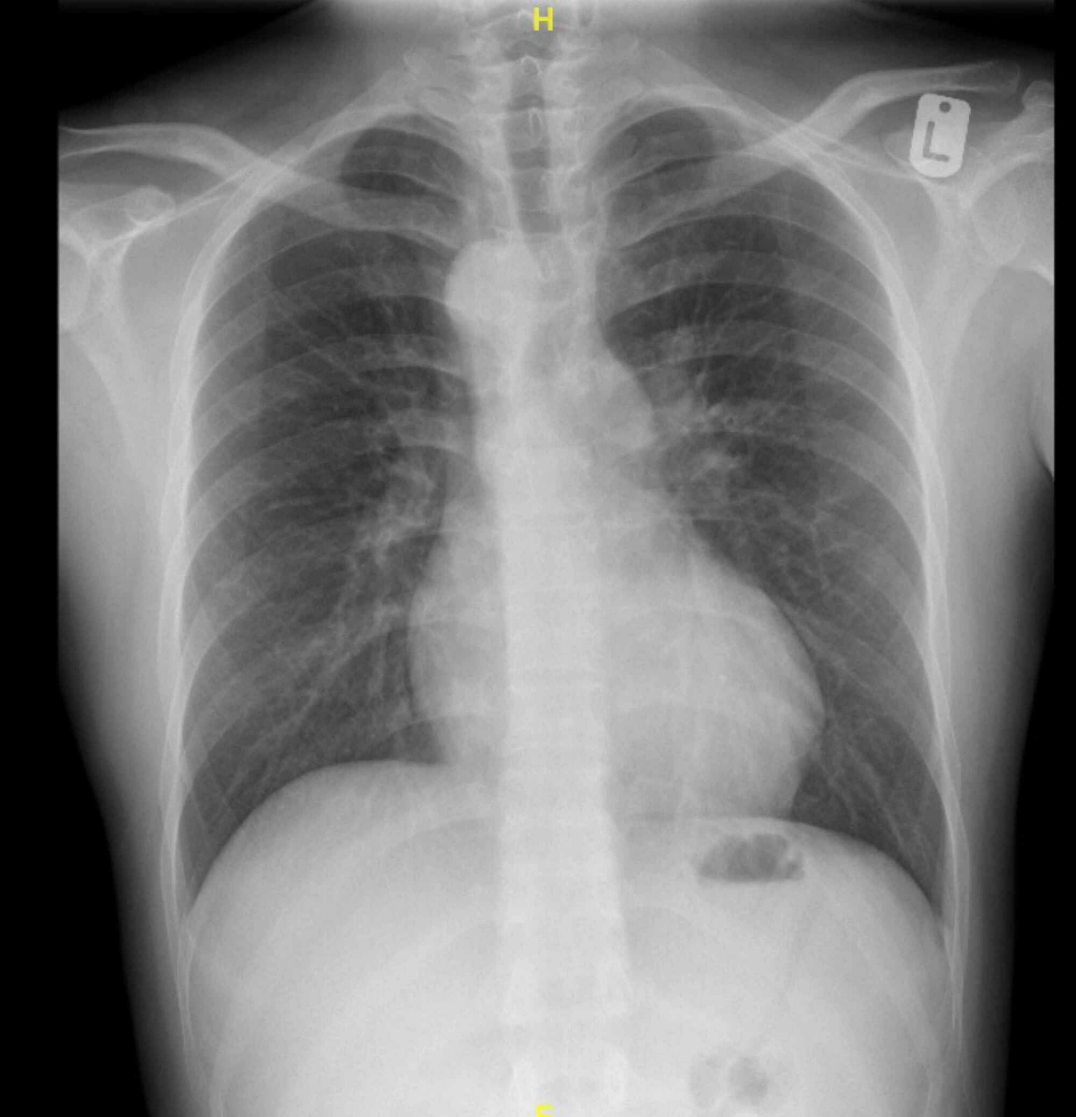
Patient’s chest X-ray demonstrating normal appearance of the heart.

CXR was also obtained and revealed a normal appearance of the heart (Figure 2).

**Figure 3:**
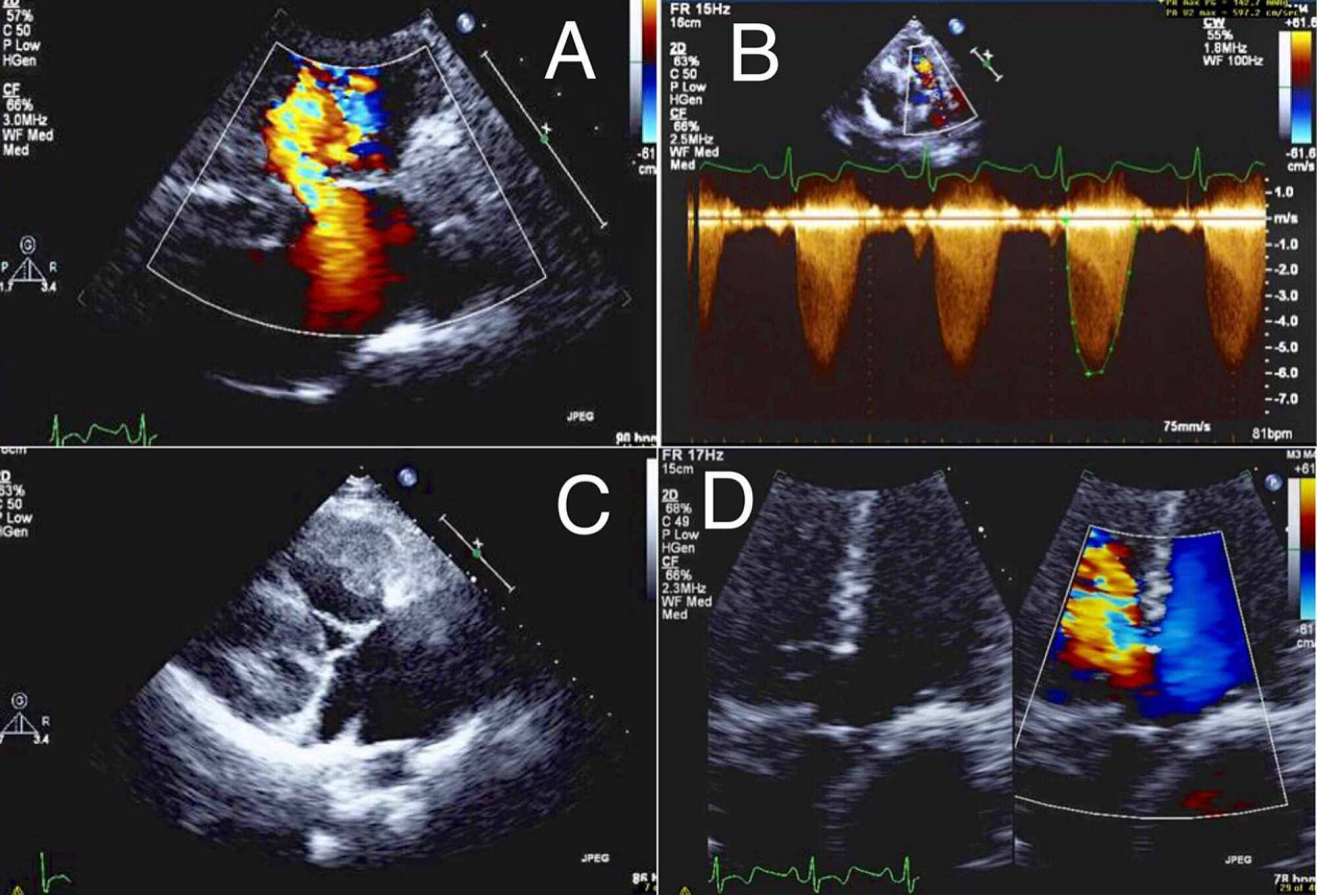
Patient’s echocardiogram showing (A) membranous ventricular septal defect with a right to left shunt, (B) severe pulmonic valve stenosis with a presence of dynamic obstruction, (C) severe right ventricular hypertrophy, and (D) overriding aorta.

An urgent echocardiogram for the patient showed a membranous ventricular septal defect (VSD) with a right to left shunt (Figure 3A), severe pulmonic valve stenosis with a presence of dynamic obstruction (Figure 3B), severe RVH with severe tricuspid valve regurgitation (Figure 3C), and overriding aorta (Figure 3D).

**Figure 4:**
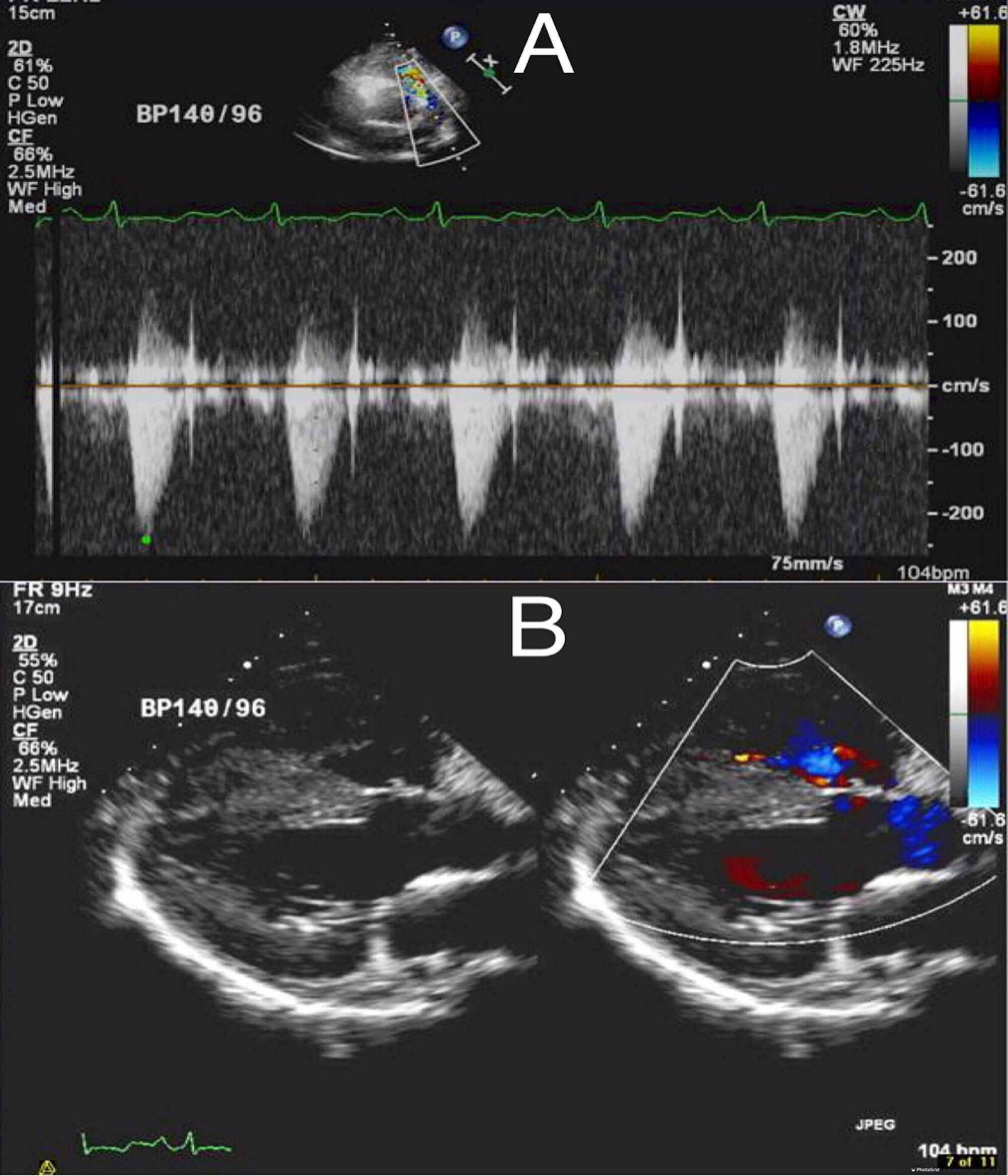
Patient’s post operational echocardiogram demonstrating (A) prosthetic pulmonary valve and (B) non-leaking ventricular septal defect closure.

The cardiac magnetic resonance image (MRI) confirmed the echocardiogram findings. After establishing the diagnosis of ToF, the patient was referred to cardiac surgery for a repair. Total correction of ToF was executed, resulting in VSD closure with a pericardium patch, pulmonic valve replacement with bioprosthetic PERIMOUNT Magna valve in addition to annuloplasty with an MC3 ring, and an intentional patent foramen ovale was performed. The patient's symptoms improved after the corrective surgery, and he was discharged on perindopril, metoprolol, and aspirin. A five-month follow up echocardiogram demonstrated similar findings as to the previous study with a satisfying prosthetic pulmonary valve and non-leaking VSD closure (Figure 4).

Two years afterward, the patient was doing well, not complaining of any symptoms, and maintaining regular daily activities without any inconvenience.

## Discussion

In this era, the diagnosis of ToF is usually achieved during childhood. When compared to 50 years ago, a review of ToF cases demonstrated that 17.6% of the studied population were more than 25 years old at the time of diagnosis [8]. Despite the diagnostic tools’ advancement and a better understanding of the condition compared with previous centuries, there are still some delayed presentations being reported. Factors that lead to ToF delayed presentations or diagnoses can be attributed to mild symptoms and lack of health care system accessibility.

Though uncorrected ToF survival is uncommon, it has been reported that around 10% of affected persons can survive to adulthood, and only 5% reach 40 years of age [9,10]. Moreover, similar to the different degrees of presenting symptoms due to anatomical variants, unrepaired ToF might be diagnosed late as survivors could have favorable anatomo-physiology that generally permits better pulmonary flow, in contrast to those who presented earlier in their life [11]. Mechanisms that explain longevity in patients who remained undiagnosed and survived to their adulthood may be attributed to having a small pulmonary artery and slow development of subpulmonary obstruction, left ventricular hypertrophy, and extracardiac shunting or systemic to pulmonary shunt [12,13]. In our case, the reason for delayed presentation is not completely clear, but it may be attributed to slow development of pulmonary valve stenosis.

Cardiac catheterization plays a crucial role in the management of ToF in adults, not to characterize the pulmonary arteries anatomically alone, but also to define unanticipated anomalies such as aortopulmonary collateral arteries which are found in 15% of ToF patients [14]. The performance of restorative procedural to similar abnormalities aids in simplifying surgical management.

Delayed diagnosis subsequently affects the timing of the intervention. Generally, patients undergoing ToF repair are expected to have excellent results [15]. Nonetheless, ToF repair during adulthood carries a higher risk of developing arrhythmias, heart failure, and sudden cardiac arrest [16]. The higher mortality in adults than the pediatric group is caused by prolonged RV dysfunction and pulmonary artery poor development, resulting in long-standing cyanosis affecting the quality of life [17,18]. 

The physical health status is not the only one affected by the delayed intervention. Adults with ToF who underwent a repair procedure tend to have significantly poor mental health [18]. The psychological effect interferes with daily life activities, but it also majorly influences the treatment plan. The burden also extent to negatively affects patients’ professional career and family members [19].

## Conclusions

We presented a case of a patient who was diagnosed to have ToF during his adulthood. The case postulates that patients with primary congenital diseases may remain undiagnosed until an older age despite medical advances. Delaying the diagnosis and management of ToF cases increases the risk of adverse outcomes. However, our patient had an excellent post-operative and two-year follow-up profile. Thorough physical examination of newborns and a screening echo in the early life may aid in detecting the disease earlier.
